# Beyond Symbiosis: Cleaner Shrimp Clean Up in Culture

**DOI:** 10.1371/journal.pone.0117723

**Published:** 2015-02-23

**Authors:** Thane A. Militz, Kate S. Hutson

**Affiliations:** Centre for Sustainable Tropical Fisheries and Aquaculture, College of Marine and Environmental Sciences, James Cook University, Townsville, Queensland, Australia; Institute of Marine Research, NORWAY

## Abstract

Cleaner organisms exhibit a remarkable natural behaviour where they consume ectoparasites attached to “client” organisms. While this behaviour can be utilized as a natural method of parasitic disease control (or biocontrol), it is not known whether cleaner organisms can also limit reinfection from parasite eggs and larvae within the environment. Here we show that cleaner shrimp, *Lysmata amboinensis*, consume eggs and larvae of a harmful monogenean parasite, *Neobenedenia* sp., in aquaculture. Shrimp consumed parasite eggs under diurnal (63%) and nocturnal (14%) conditions as well as infectious larvae (oncomiracidia) diurnally (26%). Furthermore, we trialled the inclusion of cleaner shrimp for preventative parasite management of ornamental fish, *Pseudanthias squamipinnis*, and found shrimp reduced oncomiracidia infection success of host fish by half compared to controls (held without shrimp). Fish held without cleaner shrimp exhibited pigmentation changes as a result of infection, possibly indicative of a stress response. These results provide the first empirical evidence that cleaner organisms reduce parasite loads in the environment through non-symbiotic cleaning activities. Our research findings have relevance to aquaculture and the marine ornamental trade, where cleaner shrimp could be applied for prophylaxis and control of ectoparasite infections.

## Introduction

Parasitism has long been considered the most common lifestyle on earth with virtually all living organisms serving as potential hosts [1,2]. Parasites permeate all ecosystems and trophic levels and the aquaculture environment (inclusive of aquatic holding systems) is no exception. Manipulation of several biotic and abiotic factors in aquaculture, such as food availability, population density, water quality parameters, physical handling and the absence of predation, offsets the natural ecosystem balance [3–5]. This manipulation often favours proliferation of parasites with direct life cycles, as stressors may interact with parasites, their hosts, and the host—parasite relationship in myriad ways [6,7]. Aquaculture remains the fastest primary growth industry in the world but is heavily burdened by parasitic outbreaks [8]. The complications and ramifications of traditional chemical treatments have led industry to seek natural alternatives to parasite management. Biocontrols have been proposed as a natural means to restore ecosystem balance in aquaculture and reduce parasite intensities, most often in the form of ‘cleaner’ organisms. A diversity of aquatic organisms have been identified to engage in cleaning activities [9], with cleaner fish and shrimp proving most feasible for cohabitation with aquaculture stock.

Research efforts assessing the application of cleaner organisms in aquaculture have focused exclusively on host-cleaner symbioses for removal of host-attached parasite life stages. Secondary benefits of cleaner organisms extending beyond host-attached parasite removal have been demonstrated (cleaner fish remove fouling organisms from salmon cages [10,11]) but never extended to the possibility of non-symbiotic parasite removal. However, eggs and larval stages of parasites often persist within the culture environment and may render ‘clean’ fish immediately susceptible to reinfection [12,13]. In the case of the cleaner wrasse, *Labroides dimidiatus*, the nocturnal suspension of cleaning behaviour has been demonstrated to result in a resurgence of parasite abundance in natural systems that undermine daily cleaning efforts [12]. This phenomenon is a principle problem behind reactive disease treatments targeting a single life stage of the infectious agent and could undermine the effectiveness of cleaner biocontrols in the culture environment. Consequently, assessing the extent to which cleaner organisms engage in environmental, or non-symbiotic, ‘cleaning’ is highly advantageous.

This paper aims to demonstrate that the beneficial services of cleaner organisms extend beyond symbiosis to include removal of all off-host life stages of a marine monogenean parasite. We assessed the capacity for the cleaner shrimp, *Lysmata amboinensis*, to engage in non-symbiotic cleaning behaviours under diurnal and nocturnal conditions. The impact cleaner shrimp have on infection success of a potential host fish was also evaluated.

### Experimental parasite

A *Neobenedenia* sp. isolate from Queensland, Australia, was selected as the experimental parasite. This genus of marine capsalid monogenean is problematic in warm water aquaculture worldwide because of its direct life cycle, short generation times, tolerance to a wide range of environmental conditions, and lack of host specificity [14–17]. Adult parasites attached to the body surface of the host fish graze on host epithelial tissues, causing lesions and haemorrhaging, and continuously expel eggs that drift in sea water. The eggs bear long filamentous extensions that can entangle eggs on structure [18] and facilitate retention within culture environments. Eggs hatch to release free swimming, infectious larvae (oncomiracidia) that recruit to hosts and commence development into adult parasites.

Management of *Neobenedenia* currently relies on repetitious treatment aimed at removing juvenile and adult parasites from the host. The effectiveness of such treatment regimens is often limited as monogenean eggs are notorious for their capacity to withstand the commonly employed chemical and freshwater bath treatments due to a sclerotized protein shell protecting the developing embryo [19–22]. This allows reinfection to occur immediately after treatment [13].

## Material and Methods

### Ethics statement

Animal husbandry was monitored and experimental procedures reviewed by James Cook University (JCU) Animal Ethics Committee (Approval No. A1945). Representative adult *Neobenedenia* sp. from the laboratory stock infection providing the eggs and larvae utilized in this study were accessioned to the South Australian Museum Australian Helminth Collection, Australia (SAMA AHC 35461). *Neobenedenia* sp. used in experiments were collected from private land in north Queensland, Australia. Future permissions should be directed to Coral Coast Barramundi Pty Ltd.

### Maintenance of cleaner shrimp

Twelve *L. amboinensis* were obtained from a commercial ornamental fish collector Cairns Marine, Cairns, Australia. Shrimp were initially held separately in 12, 20 L ‘holding’ tanks and fed to satiation twice daily with a commercially available crustacean pellet (INVE NRD G8 Micro Pellet). Shrimp were given two weeks to acclimate to captivity prior to experimentation.

### Exposure to eggs

To determine whether *L. amboinensis* consume *Neobenedenia* sp. eggs, shrimp were exposed to eggs under diurnal and nocturnal conditions. Twelve *L. amboinensis* were transferred to 12 separate, 2 L aquaria (effective volume 1.8 L) containing filtered seawater (35 ppt, 26°C). Each aquarium measured 16.5 x 16.5 x 9.5 cm (L x W x H) and was constructed out of white polypropylene. Aquaria were supplied with gentle aeration and contained 50–60 embryonated (< 12 h old) *Neobenedenia* sp. eggs. Only after the eggs had settled to the bottom of aquaria were shrimp introduced. To account for potential differences in diurnal/nocturnal feeding, six replicate shrimp were given a 12 h period to consume eggs under constant illumination (between the hours 0900 and 2100) and six replicate shrimp were given a 12 h period to consume eggs in darkness (between 2100 and 0900). Shrimp had no previous experimental exposure to *Neobenedenia* eggs at this point. Following the 12 h period, shrimp were removed from their treatments gently by hand to avoid the accidental extraction of any eggs and returned to their individual-specific holding aquaria. The contents of the 2 L aquaria were then examined under a dissecting microscope (60x magnification) to determine the number of viable eggs present following the 12 h exposure period.

To determine if *L. amboinensis* increased their consumption of *Neobenedenia* sp. eggs with repeat exposure, shrimp were exposed to eggs a total of ten replicate times. The above protocol was repeated to track individual performance. For all shrimp, the initial condition (i.e. diurnal or nocturnal exposure) was maintained for all ten replicates. Shrimp were given a minimum 36 h rest period (and fed) in their holding aquaria between replicates.

To control for efficiency of egg recovery, 50–60 *Neobenedenia* sp. eggs were maintained in 2 L aquaria for 12 h with no *L. amboinensis*. The disturbance caused by extracting the shrimp by hand was mimicked, and then the number of viable eggs present was counted. The control treatment was replicated 10 times.

### Exposure to oncomiracidia

To determine whether *L. amboinensis* consume *Neobenedenia* sp. oncomiracidia following hatching, shrimp, naïve to prior experimental oncomiracidia exposure, were exposed to oncomiracidia in illuminated conditions. Six *L. amboinensis* were transferred individually to the 2 L exposure aquaria described above. Each shrimp was exposed to 50 *Neobenedenia* sp. oncomiracidia (~28 L^-1^) for a 12 h period from 0900 to 2100 under fluorescent light. Shrimp were exposed to oncomiracidia only under diurnal conditions as nocturnal exposure is unlikely to occur given the hatching rhythms and lifespan of *Neobenedenia* sp. oncomiracidia [23]. Only newly hatched oncomiracidia (< 2 h old) were used in this experiment and gently transferred to aquaria using a glass pipette. After the 12 h exposure period, shrimp were returned to their holding tanks by gently removing shrimp from their experimental tanks by hand to avoid removing any surviving oncomiracidia. The contents of the 2 L exposure aquaria were then examined under a dissecting microscope (60x magnification) to determine the number of oncomiracidia not consumed by *L. amboinensis* during the 12 h period.

To determine if *L. amboinensis* increased their consumption of *Neobenedenia* sp. oncomiracidia with repeat exposure, shrimp were exposed to oncomiracidia a total of 10 replicate times. The above protocol was repeated with care made to track individual performance. Shrimp were given a minimum 36 h rest period (and fed) in their holding aquaria between replicates.

To control for oncomiracidia recovery, 50 oncomiracidia were maintained in 2 L aquaria for 12 h without *L. amboinensis*. The shrimp extraction disturbance was replicated by hand. Contents of the aquaria were counted. The control was replicated ten times.

### Infection experiment

To determine whether *L. amboinensis* could effectively prevent *Neobenedenia* sp. infection of a suitable fish host in a culture environment, *Neobenedenia* sp. oncomiracidia were exposed to female lyretail anthias, *Pseudanthias squamipinnis* (Serranidae), in the presence of shrimp. The lyretail anthias is a frequently traded commodity in the marine ornamental trade, which is negatively affected by *Neobenedenia* spp. (see [17]). Five, 20 L aquaria containing filtered seawater (35 g L^-1^, 26°C; effective volume 16 L) were setup containing a single *P. squamipinnis*, and a single *L. amboinensis*. Additionally, all tanks were provided with aeration, rock rubble (10 cm x 10 cm) and a PVC ‘hide’ of standard length (12 cm) to minimize stress on the animals. Shrimp and fish were given 18 h acclimation before introduction of 15 newly hatched (<2 h old) oncomiracidia at 0900. Shrimp and fish were maintained communally for 30 h (exceeding the average lifespan of *Neobenedenia* oncomiracidia), during which they were fed the pellet diet twice (1000 and 1700). At the end of the exposure period fish were removed, bathed in freshwater, which kills and detaches *Neobenedenia* from the host [24], and any successful *Neobenedenia* sp. recruits counted under a dissecting microscope. Immediately following bathing, fish were laid on a glass Petri dish and photographs of both lateral sides were taken to qualitatively denote changes in appearance. Five control aquaria were also run under the same experimental conditions as the treatment except in the absence of an individual *L. amboinensis*.

### Data analyses

Values for each parameter measured were expressed as the arithmetic mean ± standard error (SE). The counts of *Neobenedenia* sp. eggs and oncomiracidia consumed during exposure experiments were presented as a percentage and independently analysed using permutational multivariate analysis of variance (PERMANOVA) based on Euclidean distances with PRIMER 6 (Version 6.1.13) and PERMANOVA+ (Version 1.0.3) statistical package. Linear regressions were used to assess cleaner shrimp performance over time using STATISTICA (Version 12) statistical package. Oncomiracidia infection success was presented as percentages and analysed using PERMANOVA. Statistical differences were accepted at *P* < 0.05.

## Results

**Fig 1:**
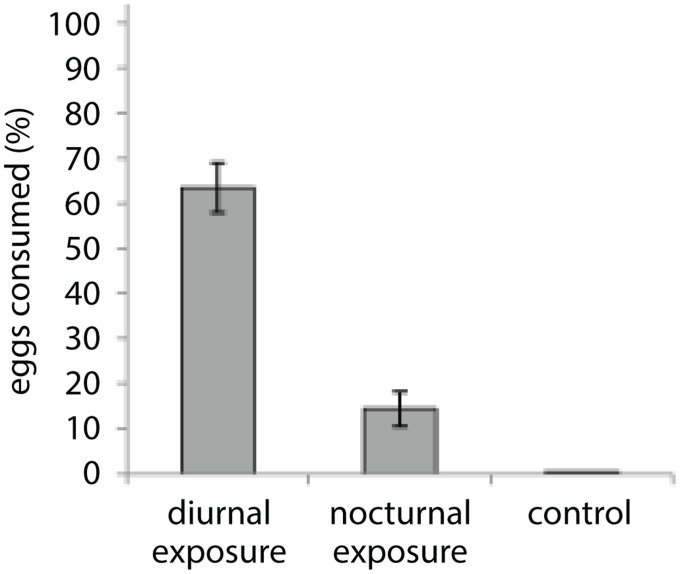
Proportion of monogenean eggs, *Neobenedenia* sp., consumed by cleaner shrimp, *Lysmata amboinensis*. Difference in mean proportion (±SE) of egg removal from the culture environment during diurnal and nocturnal exposure treatments in comparison to a handling control.

**Fig 2:**
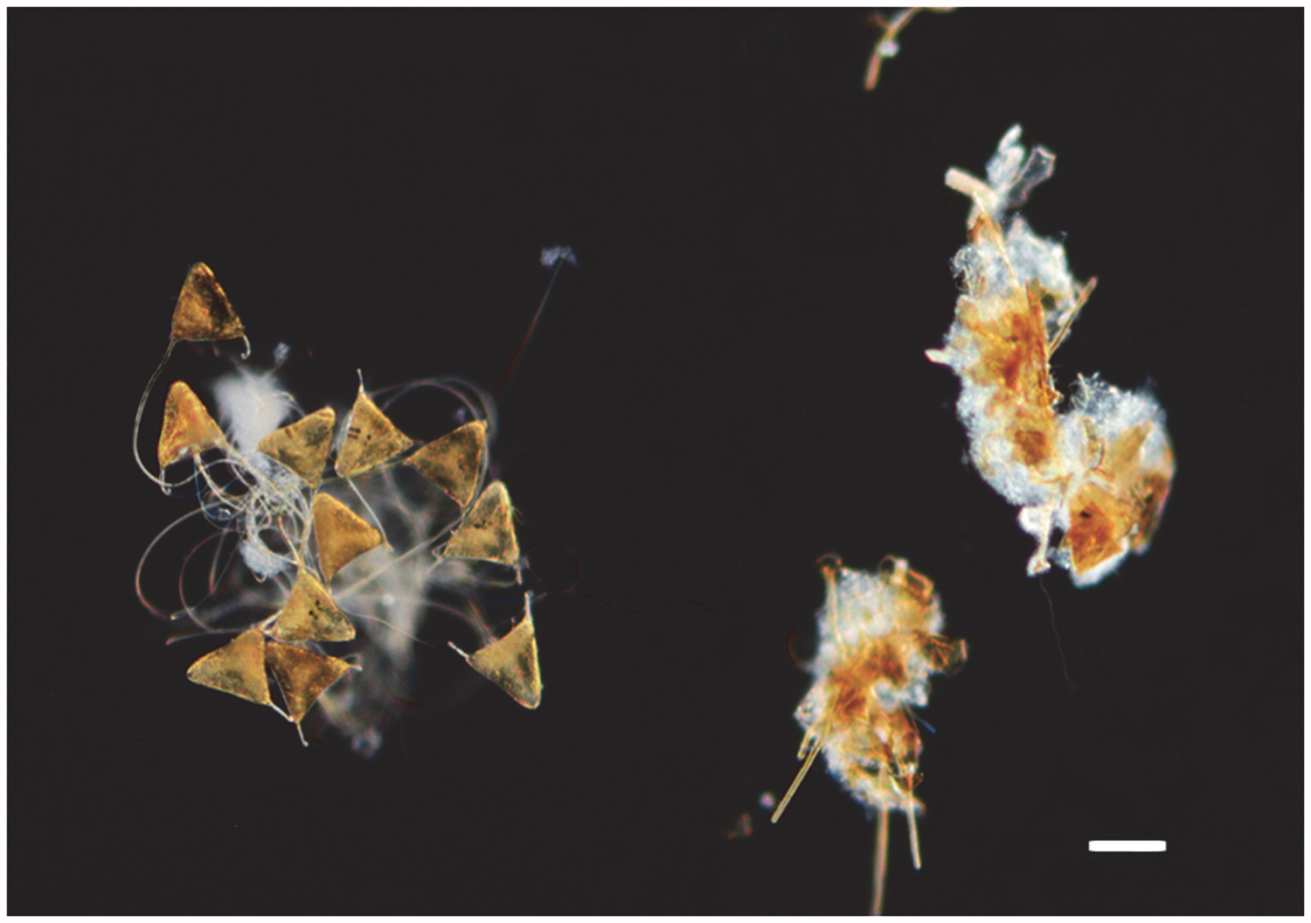
Impact of cleaner shrimp, *Lysmata amboinensis*, activity on monogenean eggs (*Neobenedenia* sp.). Eggs present in handling control (left) remained intact and viable. Eggs recovered from shrimp faeces (right) were heavily masticated and non-viable. Scale bar, 100 μm.

The cleaner shrimp, *L. amboinensis*, consumed monogenean parasite eggs and larvae from the culture environment. Under diurnal conditions, cleaner shrimp consumed two-thirds (63.5 ± 5.3%) of *Neobenedenia* sp. eggs present in aquaria during the 12 h experimental period (99.5 ± 0.3% of eggs were recovered in handling controls; Fig. 1). Clear evidence of egg consumption was apparent with masticated, non-viable egg remnants easily identified in shrimp faeces (Fig. 2). Cleaner shrimp also ate eggs under nocturnal (14.4 ± 3.8%) conditions, albeit significantly fewer compared to diurnal exposure (63.5 ± 5.3%; *F*_*pseudo*(2, 127)_ = 36.4, p < 0.05). The abundance of free-swimming larvae (oncomiracidia) was also reduced with the presence of cleaner shrimp, with shrimp eating a quarter (26.1 ± 1.8%) of all oncomiracidia (loss of 7.2 ± 1.4% in handling controls (*F*_*pseudo*(1, 62)_ = 11.1, P<0.01)).

**Fig 3:**
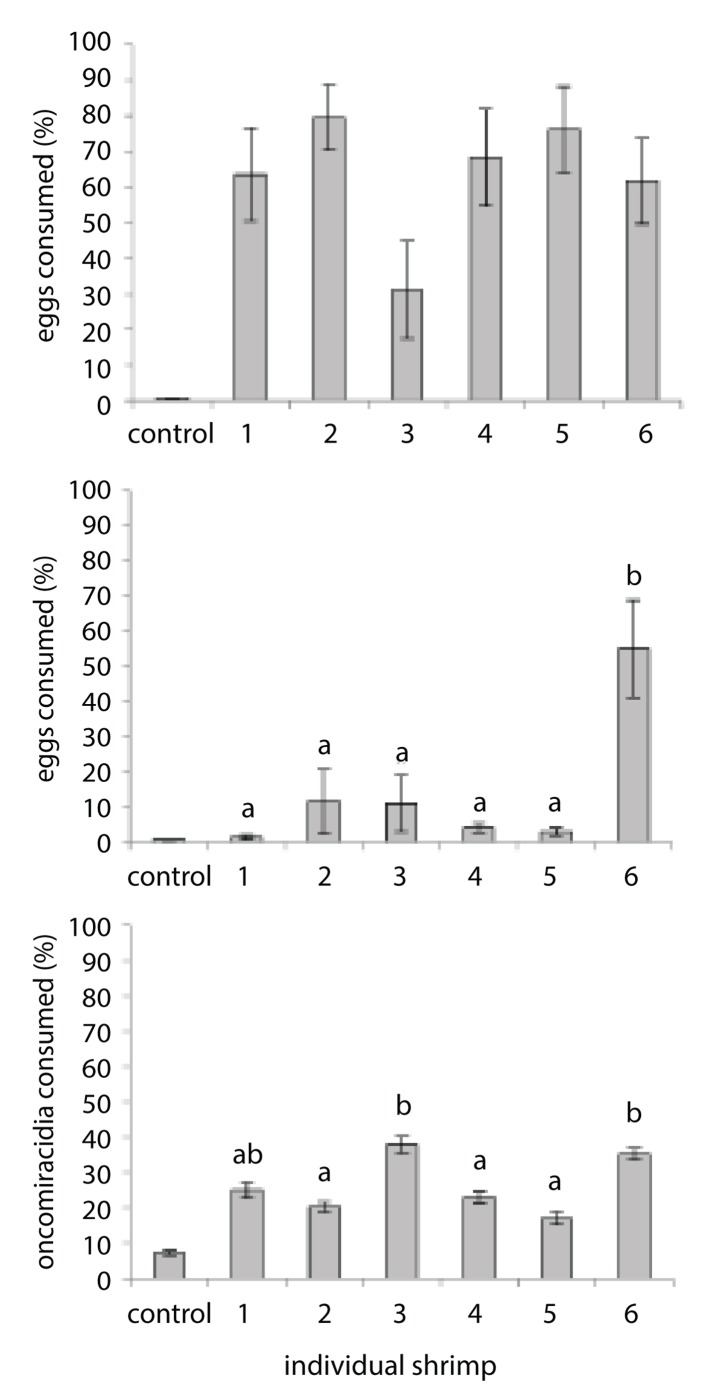
Difference in individual cleaner shrimp performance in removing eggs (a) diurnally, (b) nocturnally, and (c) oncomiracidia diurnally from the culture environment. Individuals were unique to their respective experiments and superscripts indicate statistical significance where letters are not shared. Handling control data provided for visual comparison.

Some individual cleaner shrimp performed ‘non-symbiotic cleaning’ better than others. Individual performance was significantly different among shrimp exposed to eggs in nocturnal treatments (mean range 1.4 ± 0.7 to 55.1 ±13.8%; *F*_*pseudo*(5, 54)_ = 7.4, *P*<0.01; Fig. 3) and to oncomiracidia (mean range 7.2 ± 1.4 to 37.8 ± 4.7%; *F*_*pseudo*(5, 52)_ = 4.8, *P*<0.01; Fig. 3). While there was no significant difference of egg consumption within diurnal egg exposure (mean range 31.1 ± 13.8 to 79.7 ± 9.1%; *F*_*pseudo*(5, 54)_ = 2.0, *P* = 0.09; Fig. 3), the quantity of eggs presented to shrimp was a limiting factor on the variance of individual performance with several individuals consuming all eggs during replicate exposures. Cleaner shrimp did not consume more eggs over time (diurnal eggs: *t*_(58)_ = 0.96, *P* = 0.34; nocturnal eggs: *t*_(58)_ = 0.77, *P* = 0.43; diurnal oncomiracidia: *t*_(58)_ = 0.05, *P* = 0.96).

**Fig 4:**
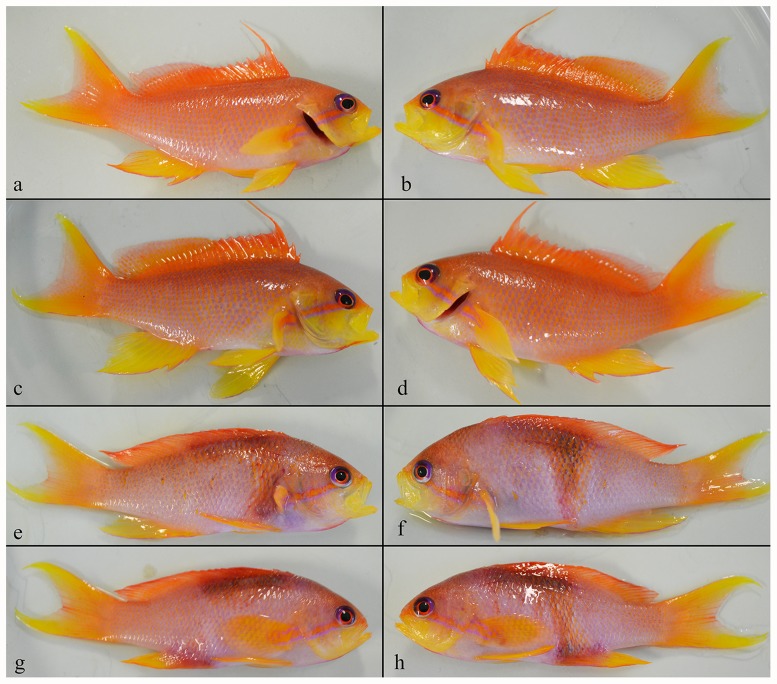
Individual *Pseudanthias squamipinnis* (7.3 ± 0.8 g) 30 h following the introduction of 15 *Neobenedenia* sp. oncomiracidia. Photos are of left and right lateral sides of two representative fish from the experimental treatment, with shrimp (a-d), and the control, without shrimp (e-h).

Cleaner shrimp reduced infection success of *P. squamipinnis* by half (25.3 ± 4.4%) compared to controls (49.3 ± 4.5%; *F*_*pseudo*(1, 8)_ = 14.4, *P*<0.02). *Neobenedenia* sp. infection prevalence was 100% for both the control and treatment, but only fish hosts maintained in absence of cleaner shrimp (control) exhibited localised dark patches and/or a general loss of epithelial pigmentation (Fig. 4e–h). Visual observations at the start of the experiment confirmed all fish were similarly pigmented.

## Discussion

To the best of our knowledge, this is the first study to demonstrate that cleaner organisms engage in non-symbiotic cleaning and consume off-host parasite life stages in their environment. Cleaner shrimp significantly reduced the abundance of eggs and larvae of *Neobenedenia* sp., a harmful, pathogenic monogenean in aquaculture and ornamental industries. In the absence of cleaner shrimp, parasitic larvae were more effective at infecting their hosts. Thus, cleaner shrimp are analogous to house pest exterminators and doctors, as they may play essential roles in preventative care and treatment [25]. Cleaner shrimp could provide a low-impact, eco-friendly alternative to parasite management in food fish and ornamental aquaculture systems compared to chemical treatments which are largely ineffective against parasite eggs and larvae [26–28]. These findings suggest that utilising the natural behaviours of cleaner shrimp may help facilitate proactive preventative management of parasitic disease in an industry almost exclusively reliant on reactive treatments.

The capacity for cleaner shrimp to masticate parasite eggs and render them non-viable is highly advantageous from a parasite management perspective. The clear sundering of monogenean eggs observed is likely attributed to the functional morphology of *L. amboinensis* mouthparts which favour mastication and cutting prior to ingestion [29]. Cleaner shrimp may prove superior to cleaner fish in non-symbiotic cleaning since qualitative observations show parasite eggs can pass through the intestinal tracts of cleaner and non-cleaner fish unabated in their capacity to hatch [30,31]. Reinfection of aquaculture stock treated for *Neobenedenia* sp. commonly occurs due to their resilience to conventional treatments and capacity for egg retention within the culture environment [13, 20–22]. This plagues the effectiveness of such treatments, and makes eradication or minimising the number of viable parasite eggs within the culture system, as demonstrated by *L. amboinensis*, highly desirable.

This is the first instance in which the nocturnal activity of cleaner organisms has been empirically demonstrated to reduce parasite loads in a system (in nature or culture). Previous research focused on cleaner fish species (*Labroides dimidiatus* and *Gobiosoma evelynae*) shows cleaners are strictly diurnal in their cleaning behaviours allowing a resurgence of parasite abundance on ‘cleaned’ host fishes the following night [12,32,33]. By continuing with non-symbiotic cleaning behaviours through the night, cleaner shrimp would continue to reduce the overall parasite load of the system and mitigate the incidence of reinfection. Cleaner shrimp were highly efficient at removing parasite eggs during the day which strongly contrasts with the premised nocturnal nature associated with *Lysmata* spp. in the natural environment [34]. Predation may be largely dependent on visual cues as evidenced by cleaner shrimp being less effective in removing eggs nocturnally and oncomiracidia, which are almost entirely transparent. Predation through visual cues has been suggested for other cleaning organisms such as the cleaner fish *Labroides dimidiatus* which is believed to target monogenean adults with poor camouflage [35]. Indeed, the reliance of cleaner organisms on visual cues may provide an evolutionary explanation for the lack of pigmentation present in the adult and free-swimming larval stages of some monogeneans [9,36,37].

Cleaner shrimp consumption rates of parasites within the culture environment did not increase with replicate exposure suggesting that cleaning behaviours are an innate rather than a learned response. While this theory has not previously been demonstrated for cleaner shrimp, Skiftesvik et al. [38] found hatchery reared cleaner fish, *Labrus bergylta*, as effective in removing ectoparasitic salmon lice (*Lepeophtheirus salmonis*) as wild caught counterparts despite no prior experience with symbiotic cleaning or association with wild fish (hosts or cleaners). However, in our study, the total number of eggs presented to cleaner shrimp may have limited the capacity for improvement in the diurnal trial with some individuals already consuming > 90% of eggs with the first replicate exposure. This may also explain the lack of a significant difference in the performance of individuals exposed to eggs diurnally as several individuals achieved maximum performance (removal of all eggs) during replicate exposures. However, a disparity in the individual performance of shrimp was clearly present for oncomiracidia and nocturnal egg exposure. The observed difference in individual performance suggests prior cleaning performance studies using small replicates of cleaner organisms (e.g.[39]) may not reflect population performance. Furthermore, as the aquaculture industry advances towards reliably producing cleaner organisms through culture [40], selection of brood stock may prove necessary to maximize the industry benefits.

Pigmentation of fish, indicative of either a stress response or tissue damage associated with infection, was dependent on treatment. Fish in the presence of cleaner shrimp where found to be infected with fewer parasites and did not exhibit the pigmented stress response observed in control fish. Changes to epithelial pigmentation are a known physiological response to stress among some teleosts [41] and often accompanies tissue damage in *Neobenedenia* sp. infections [17]. Anomalies in pigmentation can severely compromise the marketability in the ornamental and food fish trades, both of which make purchases based on aesthetic grounds [42,43]. Thus, co-habiting fish with cleaner shrimp could equate to a reduction in physiological stress for fish exposed to parasite infection and maintain product quality for sale.

## Conclusions

The success of temperate aquatic biocontrols (i.e. cleaner fish in salmon culture [10,11,44]) employed for their symbiotic cleaning services demonstrates the potential for cleaner organisms in tropical aquaculture. However, the application of tropical cleaner fish is limited by nightly suspension of cleaning activities [32,33] and the capacity for parasite eggs and larvae in the culture environment to reinfect host fish [12]. Furthermore, problems with using cleaner fish to treat client fish in aquaculture are already being realised through the potential for disease transfer from cleaner fish to clients [45]. By use of a crustacean cleaner the evolutionary distance between cleaner and client is greatly increased thereby limiting capacity for disease transfer. Here we show that the cleaner shrimp, *Lysmata amboinensis*, consumed all off-host life stages of a monogenean parasite of significant concern to aquaculture, cleaned under nocturnal conditions, and reduced infection success on host fish. The closed experimental aquaria utilized in this study mimic aquaculture systems commonly employed in the live fish and aquarium trade which can enable immediate application of these research findings. Discovery of this cleaner organism’s role in environmental pest-control greatly expands the potential efficacy of cleaner shrimp as biocontrols in food producing sectors and warrants further investigation into other secondary benefits of cleaner organisms.
